# Assessment of Ceragenins in Prevention of Damage to Voice Prostheses Caused by *Candida* Biofilm Formation

**DOI:** 10.3390/pathogens10111371

**Published:** 2021-10-23

**Authors:** Jakub Spałek, Tamara Daniluk, Adrian Godlewski, Piotr Deptuła, Urszula Wnorowska, Dominika Ziembicka, Mateusz Cieśluk, Krzysztof Fiedoruk, Michał Ciborowski, Adam Krętowski, Stanisław Góźdź, Bonita Durnaś, Paul B. Savage, Sławomir Okła, Robert Bucki

**Affiliations:** 1 Institute of Medical Science, Collegium Medicum, Jan Kochanowski University of Kielce, IX Wieków Kielc 19A, 25-317 Kielce, Poland; jspalek@ujk.edu.pl (J.S.); Stanislaw.Gozdz@onkol.kielce.pl (S.G.); Bonita.Durnas@onkol.kielce.pl (B.D.); slawomir.okla@gmail.com (S.O.); 2 Department of Otolaryngology, Head and Neck Surgery, Holy-Cross Cancer Center, Artwińskiego 3, 25-734 Kielce, Poland; 3 Department of Medical Microbiology and Nanobiomedical Engineering, Medical University of Bialystok, Mickiewicza 2C, 15-222 Bialystok, Poland; tamara.daniluk@umb.edu.pl (T.D.); piotr.deptula@umb.edu.pl (P.D.); urszula.wnorowska@umb.edu.pl (U.W.); mticv1@gmail.com (M.C.); krzysztof.fiedoruk@umb.edu.pl (K.F.); 4 Metabolomics Laboratory, Clinical Research Centre, Medical University of Białystok, 15-089 Białystok, Poland; adrian.godlewski@umb.edu.pl (A.G.); michal.ciborowski@umb.edu.pl (M.C.); adam.kretowski@umb.edu.pl (A.K.); 5 Department of Public Health, Medical University of Białystok, 15-089 Białystok, Poland; dominika.ziembicka@umb.edu.pl; 6 Department of Endocrinology, Diabetology and Internal Medicine, Medical University of Białystok, 15-089 Białystok, Poland; 7 Department of Chemistry and Biochemistry, Brigham Young University, Provo, UT 84602, USA; pbsavage@chem.byu.edu

**Keywords:** ceragenins, silicone, impregnation, voice prostheses, biofilm, *Candida* spp.

## Abstract

This study aimed to investigate the potential application of ceragenins (CSAs) as new candidacidal agents to prevent biofilm formation on voice prostheses (VPs). The deterioration of the silicone material of VPs is caused by biofilm growth on the device which leads to frequent replacement procedures and sometimes serious complications. A significant proportion of these failures is caused by *Candida* species. We found that CSAs have significant candidacidal activities in vitro (MIC; MFC; MBIC), and they effectively eradicate species of yeast responsible for VP failure. Additionally, in our in vitro experimental setting, when different *Candida* species were subjected to CSA-13 and CSA-131 during 25 passages, no tested *Candida* strain showed the significant development of resistance. Using liquid chromatography–mass spectrometry (LC-MS), we found that VP immersion in an ethanol solution containing CSA-131 results in silicon impregnation with CSA-131 molecules, and in vitro testing revealed that fungal biofilm formation on such VP surfaces was inhibited by embedded ceragenins. Future in vivo studies will validate the use of ceragenin-coated VP for improvement in the life quality and safety of patients after a total laryngectomy.

## 1. Introduction

A tracheoesophageal puncture with voice prosthesis (VP) implantation is the most effective method of voice rehabilitation among patients after a total laryngectomy (TL) [1]. The principle of this method is to restore the connection between the airways (trachea) and upper parts of the gastrointestinal tract (esophagus, pharynx, oral cavity) through the creation of a tracheoesophageal fistula with a one-way valve device inside. This allows airflow from the trachea to the esophagus and prevents esophageal content from leaking into the airways. Currently, Blom-Singer and Provox VP models are the most commonly used [2]. Since 1980, this type of VP has been based on medical-grade silicon material [3]. One of the most significant disadvantages of silicone polymer-based VPs and other medical devices is their susceptibility to colonization by fungi and bacteria [4,5,6]. *Candida* spp. colonization and biofilm formation substantially impacts VP durability. In our previous studies, we found that the four most common *Candida* species that correlated with the characteristic structural damage of the VP material (SEM, AFM) [7] were *Candida krusei, Candida albicans, Candida glabrata,* and *Candida tropicalis*. These observations have been confirmed by the studies of other authors [6,7,8].

It has been reported that it is the biofilms of the medical devices that are mostly responsible for serious infections, for example, 90% of catheter-related infections need hospitalization. Up to 70% of *Candida* spp. bloodstream infections are associated with central venous catheters [2]. However, there is no study yet that reports the correlation observed between microbial biofilm presence on voice prostheses and local infections, but there is a potential threat that colonized devices could be the origin of systemic infections in specific situations. On the other hand, the process of biofilm growth is the main reason for VP damage and deformation, which leads to its dysfunction. Leakage of the esophageal content by the tracheoesophageal fistula is the most common and potentially life-threatening dysfunction. Laccourreye et al. report that the cause for replacement was leakage through the prosthesis valve (33% of cases) and leakage around the prosthesis (27% of cases). It may lead to aspiration pneumonia and requires urgent replacement. Increased airflow resistance of the prosthesis valve results in problems with phonation and leads to replacement (40% of cases) [9]. The average VP lifespan is variable within the range of between approximately 4 and 8 months [7,10]. The lifespan of the Provox VP may be longer in some single extremal cases [11]. There have been some attempts to manage the short effective lifespan of VP. Provox has developed a new type of valve mechanism in their devices, the Provox ActiValve, based on Provox 2. It is made of the same medical-grade silicone, but the valve seat and the valve flap are made of fluoroplastic, using magnets available in three different strengths to support valve closure. This modification has significantly elongated the VP lifespan, but the cost of the devices is high, so it is not commonly used. Moreover, the Provox ActiValve was designed for patients with frequent central-type leakage. There is no improvement in patients with the VP lateral leakage throughout the fistula [10]. There have also been other approaches to improve VP durability by reducing the development of biofilm. The application of nystatin, antimicrobial agents, oral wash, or VP cleaning by a special brush have advantages and disadvantages; however, these have no clinically satisfactory results [12,13], potentially because of insufficient candidacidal activity of the cleaning products. 

Ceragenins (CSAs) are amphiphilic derivatives of bile acids with covalently attached amines that mimic the amphipathic properties of endogenous antimicrobial peptides (AMP) expressed in many organisms including bacteria, fungi, plants, insects, worms, and mammals. The mechanism of their antimicrobial action involves membrane binding and insertion that induces morphological changes in the structure of the pathogen’s membrane leading to its destruction and cell death. Previous studies report antimicrobial activities of different ceragenins (e.g., CSA-13, CSA-131, CSA-192) against Gram-negative and Gram-positive bacteria, *Candida* species, parasites, and some viruses [14,15,16,17,18,19,20,21]. Ceragenins have better antifungal and antibacterial properties against monomicrobial or fungal–bacterial multispecies biofilms than other commonly used antimicrobials [22]. They also inhibit the attachment and the formation of *Candida* biofilms [23], and this effect can be potentiated by nanosystems containing ceragenins and metallic nanoparticles [21]. In this study, we describe the activity of CSAs against clinical isolates of *Candida* strains collected from VPs of patients after a total laryngectomy. Additionally, we also tested whether CSAs induced resistance in *Candida* over 25 passages. Overall, our observation provides strong support for the development of this group of molecules for the prevention of VP destruction associated with *Candida* colonization and biofilm development. 

## 2. Results

### 2.1. Ceragenin CSA-131 Displays the Strongest Candidacidal Activity against Tested Candida Strains from Group of Tested Antimicrobial Agents

**Figure 1:**
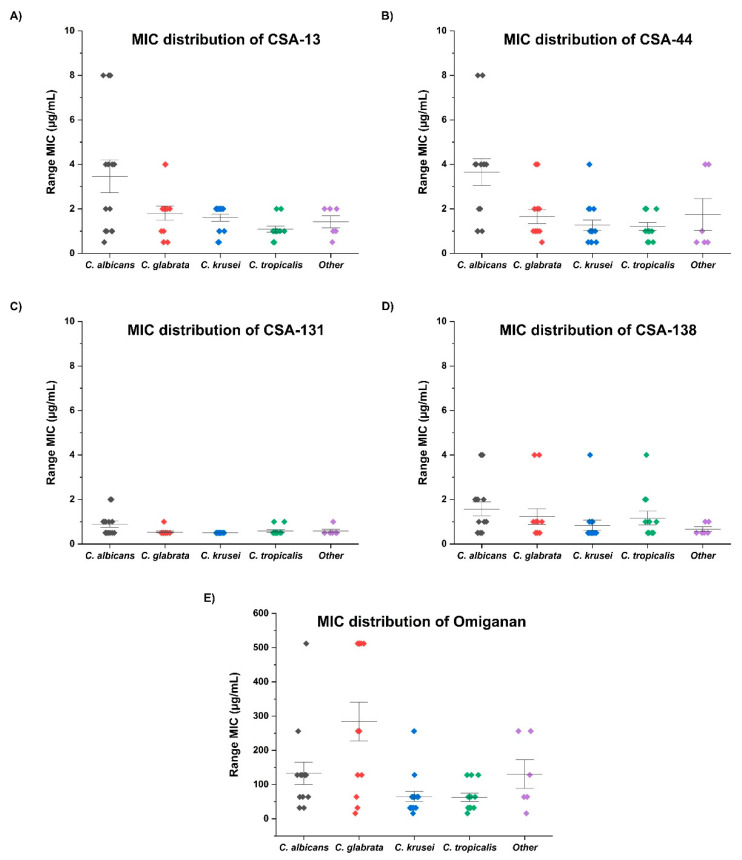
MIC distribution of tested ceragenins (**A**–**D**) and omiganan (**E**) against 60 isolates of *Candida* species.

For the first step of the study, we assessed the fungicidal activity and inhibition of biofilm formation of commonly used or currently developed antifungal agents including amphotericin B, fluconazole, and omiganan against clinical isolates of four selected *Candida* species. We also assessed the fungicidal activity of cathelicidin LL-37 and synthetic non-peptide analogs: CSA-13, CSA-131, CSA-138, and CSA-44. The results were dependent on *Candida* species, but overall, CSA-131 was the most effective antifungal molecule. The results of the antimicrobial activities of tested agents for the studied clinical isolates (*C. albicans, C. krusei, C. tropicalis, C. glabrata*) are presented in Table 1. The strongest biological activity was observed against the strains of *C. glabrata* and *C. krusei*; MIC_90_/MFC_90_ were measured at 0.5 µg/mL of CSA-131. MIC_90_/MFC_90_ of amphotericin B for tested strains was recorded at a concentration of 4 µg/mL, while the activity of cathelicidin LL-37 was significantly lower (MIC_90_/MFC_90_ = 512 µg/mL). The minimum biofilm inhibitory concentration (MBIC_90_) of CSA-131 was significantly lower than other agents with each clinical strain. The strongest antifungal activity was observed against *C. krusei* and *C. tropicalis* (1 µg/mL). Notably, MBIC_90_ for amphotericin B was the lowest among those strains as well, at a concentration of 4 µg/mL. It is noteworthy that the fungicidal activity of fluconazole and LL-37 were the lowest for each of the *Candida* strains; MIC_90_/MFC_90_/MBIC_90_ values reached 512 µg/mL. On the other hand, ceragenins were the most effective among all tested agents, and CSA-131 was the most effective among all the tested ceragenins. The MIC distribution of all tested ceragenins in different *Candida* species is presented in Figure 1.

### 2.2. Prolongated Incubation of Candida with Ceragenins Did Not Result in Development of Candida Resistance

**Figure 2:**
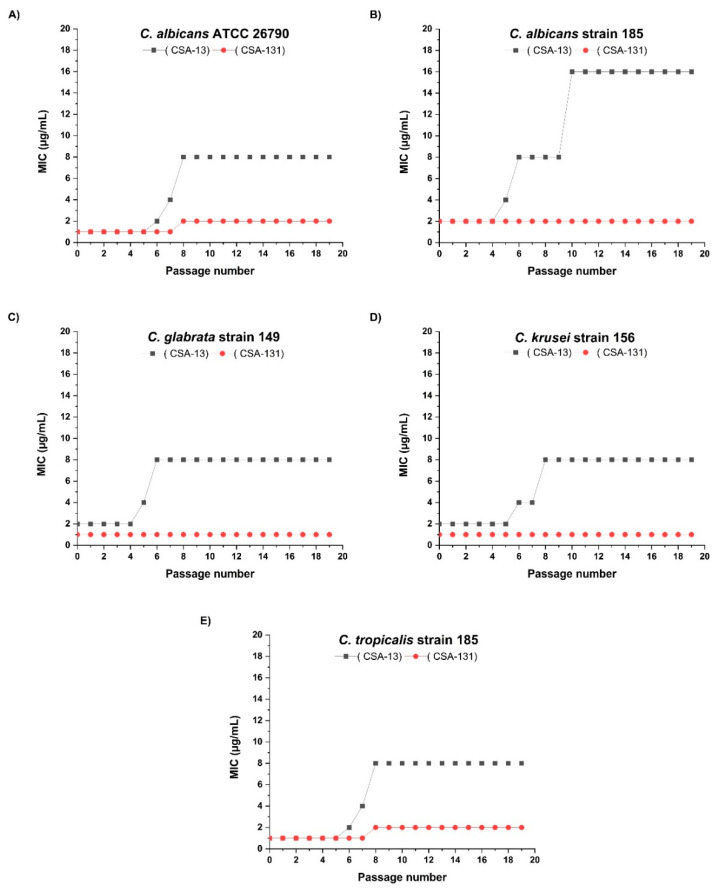
Development of resistance for ceragenins CSA-13 and CSA-131 in selected *Candida* species (**A**–**E**).

*Candida* spp. were tested in vitro for the potential development of drug resistance against ceragenins CSA-131 and CSA-13. For CSA-13, a gradual increase in MIC was observed between 4 and 6 passages with each different clinical isolate of *Candida*. However, this increase for 4 out of 5 tested strains was not above the 2-fold dilution factor, which is considered as within the range of possible error for MIC methods. In the case of CSA-131, we did not observe any increase in MICs throughout 25 passages for three isolates. With one isolate of *C. tropicalis* and one isolate of *C. albicans,* the CSA-131 MIC changed from 1 to 2 µg/mL after 7 passages (Figure 2).

### 2.3. The Impregnation of VP in an Ethanolic Solution of Ceragenin Prevents the Development of Candida Biofilm on Their Surface

**Figure 3:**
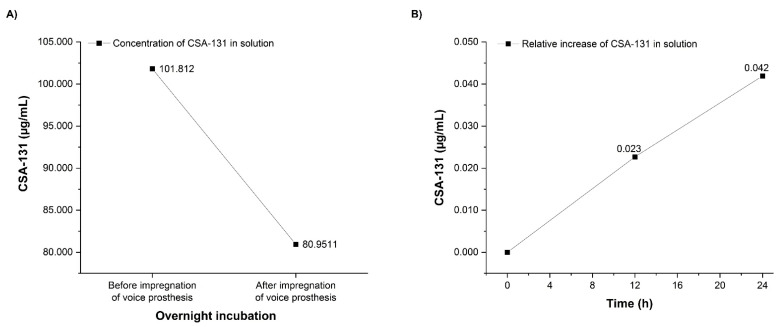
Reduction of CSA-131 concentration in solution as the result of incubation with voice prostheses (**A**). Relative release of CSA-131 from impregnated voice prostheses into solution over 24 h of incubation, calculated as the increase in the CSA-131 concentration in relation to its level measured immediately after dipping prostheses in the solution (time 0) (**B**).

Silicones are known to swell in the presence of alcohols, and this process may be used to incorporate actives into the silicone. To assess if ceragenin dissolved in isopropyl alcohol might be used to impregnate silicon, we monitored the concentration of CSA-131 in a solution incubated with a silicone VP. We observed a decrease in CSA-131 concentration after the incubation of VPs indicating a preferential interaction of the ceragenin with silicone. As presented in Figure 3, the concentration of CSA-131 in solution after VP incubation decreased by 21%. Additionally, after placing VPs infused with CSA-131 into a ceragenin-free alcohol solution, the concentration of CSA-131 in this solution slowly increased (Figure 3).

**Figure 4:**
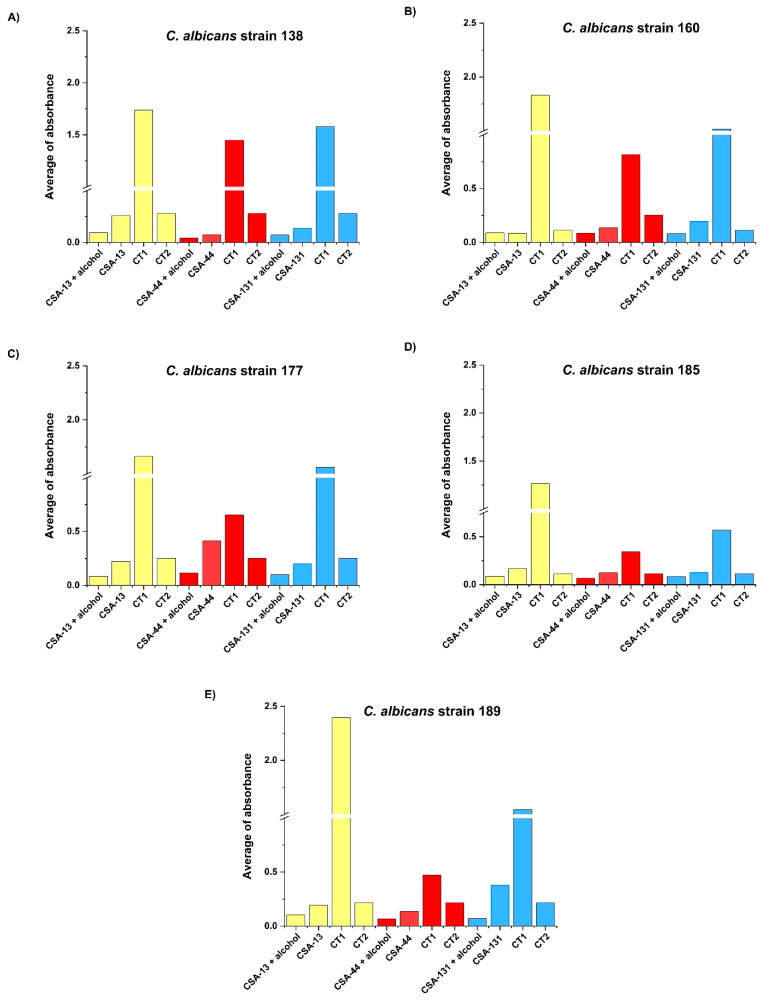
Estimation of biofilm formation by five clinical isolates of *C. albicans* (**A**–**E**) on voice prostheses within 24 h in RPMI medium initially impregnated by ceragenins: CSA-13 (yellow), CSA-44 (red), and CSA-131 (blue) with alcohol; in the presence of tested CSAs; without ceragenins (CT1) and in alcohol without tested ceragenins (CT2).

In vitro tests revealed that the fungal biofilm formation of clinical isolates of *C. albicans* was significantly inhibited on the surface of VP that were previously incubated in CSA-131 and alcohol solution versus the control groups. The study has shown that other ceragenins, such as CSA-13 or CSA-44, embedded into the silicon of VP also had fungal biofilm formation inhibition properties (Figure 4) (Table 2).

## 3. Discussion

Microbial colonization is one of the most significant disadvantages of indwelling medical devices made of synthetic materials, especially those made of silicone. Colonization presents serious clinical problems for patients with urological catheters, endotracheal tubes, intravascular catheters, orthopedic implants, and optical lens’, among others. The initial adherence of *Candida* spp. to the silicone polymer surface is likely preceded by bacteria, and the synergistic interaction between bacteria and fungi in a mixed biofilm during the surface colonization of medical devices has been described [24]. Colonizing fungi develop a biofilm on the VP surface that is responsible for VP destruction. This could also be a potential cause of local and general infections [25,26]. It is worth noting that in the case of VP, microbial colonization is promoted by the properties of the trachea–esophageal fistula environment. VPs are placed in a non-sterile niche, so microorganisms rapidly colonize them, and because of the permanent contact with the exterior environment, they are continuously exposed to additional organisms. Moreover, the microenvironment of the esophagus and trachea (presence of food, moisture, poor drug penetration) promotes biofilm formation. The volume and quality of the biofilm on VPs is different in the esophageal in comparison to the tracheal phalange [7]. This difference is likely caused by the more favorable growing conditions in the esophageal fistula. 

Multiple approaches have been evaluated for the inhibition of VP deterioration [27], including the use of probiotics [28]. Notable attempts at inhibiting biofilm growth on VPs were to change the physicochemical properties of the material making the device. However, this approach is complicated by the specific requirements for VPs. They need to maintain their flexibility and compatibility with the host tissue and to be easily inserted or replaced. On the other hand, material that is too flexible can lead to leakage or displacement in the fistula. Changing the material properties to be more resistant to destruction by biofilm growth may result in the loss of these criteria. Currently, only the reduction of silicone surface roughness and the application of anti-adhesive polymers such as 2-(dimethyl-amino)ethyl methacrylate have been shown to decrease biofilm formation in VPs [29,30,31]. Another approach is to develop surface coatings of, or impregnate the device with, antimicrobial and/or antiadhesive agents. Recently, results were reported of medical-grade silicone coated with sophorolipids for anti-adhesive and anti-microbial properties [32,33]. Tsikopoulos et al., in a meta-analysis study, have reviewed 33 comparative studies from 1999 to 2019 describing all reported in vitro attempts at inhibition of biofilm formation on silicon rubber VPs [34]. None of these approaches met the criteria of protecting VPs for extended periods without the risk of the emergence of drug resistance.

There are many agents with antimicrobial activities against *Candida* species. From commonly used antifungals such as nystatin, fluconazole, and amphotericin through endogenic and synthetic antimicrobial peptides to magnetic nanoparticles and photodynamic therapy. All of these have antimicrobial activity against *Candida* species forming mixed biofilms [2]. Some studies have assessed the antimicrobial activity of ceragenins in some medical device applications. In 2013, one study reported that CSA-138 covalently attached to the hydrogel optic lens-displayed antimicrobial activity and provided extended lifespan to the device [35]. Hashemi et al. have found, in preclinical studies, that a ceragenin-coated endotracheal tube had substantial antimicrobial activities against some *Candida* species [36]. Other studies showed significant inhibition of biofilm formation on orthopedic implants coated with ceragenins [37,38]. However, there are no studies describing the application of ceragenins or nanoparticles in the fight against specific strains of fungal isolates identified on VPs. 

In this study, we investigated the fungicidal activity of classic agents compared to ceragenins and their potential application as fungicidal agents against the most common *Candida* species isolated from biofilm residing on the damaged VPs. Additionally, we investigated the potential of the application of CSA-131 on the surface of the VP biomaterial to prevent its colonization.

This study has shown that among the ceragenins, CSA-131 is the most effective agent against the four most common *Candida* species responsible for VP deterioration (Table 1 and Figure 1). Moreover, the development of resistance for CSA-131 by these clinical isolates was not observed during 25 passages. CSA-131 also had the most effective impact on the inhibition of the mass growth of biofilm (Figure 2). We showed that the incubation of VPs in an organic solution of CSA-131 allowed impregnation of the VP with the active agent. Embedded CSA-131 showed significant antimicrobial effects in reducing the biofilm mass of *C albicans* on the VP surface in vitro. The rate and duration of the CSA-131 released from impregnated VPs were observed for 24 h. The rate was nearly linear over this period, and if this rate remained constant there is the possibility that CSA-131 would remain on the VP for approximately 1.3 years. Because of the short time of measurement, the duration of release was not determined and is the subject of further research and the use of CSA-131 for VP impregnation will require additional study. Nevertheless, a CSA-131-based disinfectant could be developed for regular VP treatment similar to that provided by Provox Flush accessories. It seems that cleaning procedures using CSA-131 could be a more effective substitute for the water recommended for this procedure by the manufacturer.

Our results demonstrate the possibility of developing a method to extend the life of VPs by increasing their resistance to the destructive effects of the most common *Candida* species with the use of cerulenin CSA-131. With the effective use of this antimicrobial, the replacement procedure could be less frequent, and the potential risk of life-threatening complications would be lower. This fact is significant because total laryngectomy is still the most effective treatment for locally advanced laryngeal cancers [39]. Laryngeal cancer is diagnosed annually in approximately 177,000 patients worldwide [40,41]. Most of these are diagnosed at stage ≥ III and many of these patients will become VP users.

## 4. Materials and Methods

### 4.1. Collection of Candida Strains

A group of 60 clinical isolates of the most common yeasts from damaged Provox VPs collected during their replacement were used in this study including 14 *Candida albicans* isolates, 15 *Candida krusei* isolates, 12 *Candida tropicalis* isolates, and 13 *Candida glabrata* isolates, 3 *Saccharomyces cerevisiae* isolates, 1 *Candida parapsilosis* isolate, 1 *Candida kefyr* isolate, and 1 *Candida dubliniensis* isolate. VPs were removed from laryngectomized patients of the Holy Cross Cancer Center. The replacement procedures were performed by physicians using sterile instruments. Directly after removal, the VPs were placed into sterile containers and immediately transported, at RT (room temperature), to the microbiology laboratory for further analysis. VPs were suspended in 5 mL of thioglycolate broth and vortexed for 2–3 min. Then, 50 µL of the eluted material was seeded onto Sabouraud Agar with antibiotics and Chromogenic Agar (all microbial media were from Thermo Fisher Scientific) for preliminary identification and incubated for 48 h at 30 °C. After incubation, yeasts were identified using Yeast ID cards (Vitek 2 automated system, bioMerieux). Identified *Candida* strains were stored in the MAST CRYOBANK system (Mast Diagnostica) at −70 °C. The stored strains were revived on Sabouraud Agar for further studies.

### 4.2. Antifungals, Ceragenins, and Determination of MIC, MFC, and MBIC 

Minimal inhibitory concentrations (MICs) were determined for amphotericin B and fluconazole (purchased from Pol-Aura, Poland), omiganan and LL-37 (purchased from LipoPharm, Gdańsk, Poland), and ceragenins CSA-13, CSA-131, CSA-138, and CSA-44 (synthesized as previously described) [42], using the microdilution method described in the guidelines of the Clinical Laboratory Standards Institute (CLSI) [43]. Antifungal activity of the tested agents against clinical isolates of *C. albicans*, *C. krusei*, *C. tropicalis*, and *C. glabrata* was determined using pathogen cells in log-phase growth. *C. albicans* ATCC 26790 and *f* ATCC 6258 were used as reference strains. Dilutions of tested compounds were prepared in Mueller–Hinton Broth (Thermo Fisher Scientific, Waltham, MA, USA) in concentrations ranging from 512 µg/mL to 0.5 µg/mL. MICs were determined visually as the lowest concentration of tested agents that showed no microbial growth after 24–48 h of incubation. In the next step, minimal fungicidal concentrations (MFCs) of all tested agents against all tested isolates were determined by inoculating the tested samples on Sabouraud dextrose agar plates, followed by incubation at 35 °C in sealed plastic bags to prevent drying. A growth control culture without antifungals was submitted to the same procedures. MFCs were determined visually as the lowest concentration of tested agents that showed no fungal growth after 24–48 h. Minimum biofilm inhibitory concentration (MBIC) was assessed using an MTT test based on the reduction of tetrazolium salts as previously described [44].

### 4.3. Subsequential Passages of Selected Candida Strains and the Assessment of Their Susceptibility to Ceragenin

The in vitro method of serial passage was used to evaluate the probability of developing resistance to ceragenin CSA-13 and CSA-131 in selected *Candida* species from all the study groups [45]. Before starting this study, the MIC values for each studied species were assessed. For this experiment, the isolate from each studied *Candida* strain with the highest value of MIC was selected. The *C. albicans* ATCC 26790 strain was used as a reference. Serial passaging was performed using Sabouraud dextrose agar plates incubated at 35 °C with the concentration of tested ceragenins just below the MIC value. After an 18–24 h incubation period, cells growing in the highest concentration on the antimicrobial from the previous passage were once again harvested and assayed for the MIC. The process was repeated 25 times.

### 4.4. Voice Prosthesis Incubation in Organic Solution of Ceragenin

The ability of ceragenins to impregnate vocal prostheses was examined after the immersion of small prosthesis fragments (3 × 3 mm) in a 10% solution of CSA-131, CSA-13, and CSA-44 in isopropyl alcohol, followed by incubation at 37 °C overnight (the CSA-impregnated group). Next, the solution was removed by pipetting and samples were placed into a vacuum chamber at 50 mbar for 4 h at room temperature to completely evaporate the remaining alcohol. At the same time, another set of the prosthesis fragments was incubated with isopropyl alcohol only and subsequently subjected to the same drying procedure (the alcohol-impregnated group), serving as the control group. All experiments were performed in triplicate.

### 4.5. Evaluation of Biofilm Mass

Five fluconazole-resistant *C. albicans* clinical isolates (Table 2) were selected to compare biofilm formation on voice prostheses (i) impregnated with a 10% solution of ceragenins in isopropyl alcohol (the CSA-impregnated group), (ii) incubated with isopropyl alcohol only (the alcohol-impregnated group), (iii) non-impregnated but treated with free ceragenins at a concentration of 2 × MBIC value (the CSA-treated group) (Table 2), and (iv) using non-impregnated and non-ceragenin-treated ones as the control group. 

The biofilm cultures were carried out in 96-well microtiter plates in 200 µL of RPMI medium (Sigma-Aldrich, Saint Louis, MO, USA) under aerobic conditions for 48 h at 37 °C. After incubation, the planktonic cells were carefully removed and the biofilms were washed twice with PBS, dried at room temperature, and stained for 15 min using 150 µL of 0.1% crystal violet (Chempur, Poland). Next, the excess of stain was removed, and the biofilms were rinsed with deionized water and the plates left to dry. To solubilize the crystal violet, 100 µL of 98% ethanol was added to each well, and the optical density (OD) was determined at a wavelength of 570 nm using the Varioscan Lux microplate reader (Thermo Fisher Scientific, Waltham, MA, USA) to estimate the number of bacteria in a biofilm. 

### 4.6. CSA-131 Quantitation

CSA-131 concentration was determined using ultra-high performance liquid chromatography (1290 Infinity II, Agilent Technologies) coupled with tandem mass spectrometry (6495 Triple Quad, Agilent Technologies) equipped with iFunnel technology. A sample (2 µL) was injected into the chromatographic column (Zorbax RRHD Eclipse Plus C18 (2.1 mm × 50 mm, 1.8 µm) with a Zorbax Eclipse Plus C18 (2.1 mm × 5 mm, 1.8 μm) precolumn; both Agilent Technologies) and thermostated at 45 °C. The flow rate was 0.5 mL/min with solvent A (deionized water with 0.1% formic acid) and solvent B (acetonitrile with 0.1% formic acid). The gradient started at 5% phase B and rapidly increased to 80% over 0.4 min, followed by an increase in phase B to 90% for another 6.6 min, remaining at this solvent ratio for 2 min. Next, the gradient changed to the starting conditions (in 0.01 min) and remained at 5% of phase B for 1.5 min. Analysis was performed in the positive (ESI+) ion mode. The single ion monitoring (SIM) mode was used. The parent ions of the mass 901.6 (M + H) and 923.7 (M + Na) were isolated by the second quadrupole. The capillary voltage was set to 3500 V and the gas temperature to 280 °C with a flow rate of 15 L/min. The nebulizer gas pressure was set at 25 psi and the sheath gas temperature at 350 °C with a flow rate of 12 L/min.

CSA-131 was derivatized using acetic anhydride. 100 µL of diluted CSA-131 solution was evaporated in the SpeedVac Concentrator (Savant SPD2010, Thermo Fisher Scientific), followed by derivatization with 50 µL of acetic anhydride. The samples were vortex-mixed and then incubated for one hour at 45 °C. The reaction mixture was then dried in the SpeedVac Concentrator. The residues were dissolved in a 100 µL mixture of water/acetonitrile 1:1 with 0.1% formic acid, and then samples were mixed for 5 min. 

### 4.7. Statistical Analysis

The significance of differences was determined using the two-tailed Student’s *t*-test. Statistical analyses were performed using Statistica 10 (StatSoft Inc, Tulsa, OK, USA). *p* < 0.05 was considered to be statistically significant. 

## 5. Conclusions

Of the tested ceragenins, CSA-131 showed the strongest activity against *C. albicans, C. krusei, C. tropicalis,* and *C. glabrata,* frequently identified as biofilm-residing fungi in biofilm growing on the surface of VPs. CSA-131 has the potential to reduce *C. albicans* biofilm mass on voice prostheses in vitro. The impregnation of VPs with CSA-131 might provide a technical approach to develop VP devices that are more resistant to *Candida* colonization. The development of a CSA-131 solution that could potentially be used as a flushing fluid for the regular maintenance of VP is also strongly supported. 

## Figures and Tables

**Table 1:** Comparative antimicrobial activities of tested agents in vitro, against four most common species of yeast isolated from damaged voice prostheses.

Agent	MIC (µg/mL)			MFC (µg/mL)			MBIC (µg/mL)		
	Range	50%	90%	Range	50%	90%	Range	50%	90%
	*Candida albicans* (n = 14)								
Amphotericin B	0.5–512	0.5	1	0.5–512	0.5	1	0.5–512	1	32
Fluconazole	0.5–512	16	512	1–512	128	512	2–512	128	512
Omiganan	32–512	128	256	64–512	128	256	64–512	128	256
LL-37	16–512	512	512	64–512	512	512	64–512	512	512
CSA-13	0.5–8	2	8	1–8	4	8	2–16	4	8
CSA-131	0.5–2	0.5	2	0.5–2	0.5	2	0.5–2	1	2
CSA-44	1–8	4	8	1–8	4	8	1–8	4	8
CSA-138	0.5–4	1	4	0.5–4	1	4	0.5–8	1	4
	*Candida krusei* (n = 15) *								
Amphotericin B	0.5–128	1	4	0.5–256	2	8	0.5–256	2	4
Omiganan	16–256	64	128	32–512	64	128	32–256	128	256
LL-37	16–512	512	512	16–512	512	512	16–512	512	512
CSA-13	0.5–2	2	2	0.5–2	2	2	0.5–8	2	4
CSA-131	0.5	0.5	0.5	0.5	0.5	0.5	0.5–1	0.5	1
CSA-44	0.5–4	1	2	0.5–4	1	2	0.5–4	1	4
CSA-138	0.5–4	0.5	1	0.5–4	0.5	2	0.5–4	1	2
	*Candida tropicalis* (n = 12)								
Amphotericin B	0.5–8	1	4	0.5–16	2	8	0.5–16	2	4
Fluconazole	1–512	2	32	2–512	4	128	2–512	4	128
Omiganan	16–128	32	128	16–256	64	128	32–256	64	128
LL-37	128–512	512	512	128–512	512	512	512	512	512
CSA-13	0.5–2	1	2	0.5–4	1	4	0.5–4	1	4
CSA-131	0.5–1	0.5	1	0.5–1	0.5	1	0.5–2	0.5	1
CSA-44	0.5–2	1	2	0.5–4	1	2	1–2	1	2
CSA-138	0.5–4	0.5	2	0.5–4	1	2	0.5–4	1	2
	*Candida glabrata* (n = 13)								
Amphotericin B	0.5–16	1	4	0.5–16	2	4	0.5–128	2	16
Fluconazole	2–512	128	512	4–512	256	512	4–512	512	512
Omiganan	16–512	256	512	32–512	256	512	64–512	256	512
LL-37	16–512	512	512	64–512	512	512	64–512	512	512
CSA-13	0.5–4	2	4	0.5–4	2	4	0.5–8	2	4
CSA-131	0.5–1	0.5	0.5	0.5–1	0.5	0.5	0.5–4	1	2
CSA-44	0.5–4	1	4	0.5–4	2	4	1–8	2	4
CSA-138	0.5–4	1	4	0.5–4	1	4	0.5–4	1	4

* *Candida krusei* exhibits natural resistance to fluconazole. MIC/MFC/MBIC values have not been determined.

**Table 2:** Antifungal activity of the tested compounds against clinical isolates of *Candida albicans* (n = 5).

	Compound	Amphotericin B	Fluconazole	Omiganan	LL-37	CSA-13	CSA-131	CSA-44	CSA-138
Strain		MIC/MFC/MBI							
*C. albicans* 185		256/256/256	256/>256/>256	128/128/128	>256/>256/>256	1/1/4	2/2/2	4/4/4	2/2/2
*C. albicans* 177		0.5/0.5/1	>256/>256/>256	64/128/128	>256/>256/>256	2/2/2	0.5/0.5/.5	1/1/1	0.5/0.5/.5
*C. albicans* 189		0.5/0.5/0.5	>256/>256/>256	128/128/128	>256/>256/>256	1/2/2	0.5/0.5/.5	2/2/4	1/1/1
*C. albicans* 138		>256/>256/>256	>256/>256/>256	>256/>256/>256	>256/>256/>256	4/4/4	1/1/1	4/4/4	1/1/1
*C. albicans* 160		1/2/2	128/>256/>256	32/64/128	>256/>256/>256	2/2/8	0.5/0.5/1	1/2/2	0.5/0.5/1

## Data Availability

The data presented in this study are available on request from the corresponding author. The data are not publicly available due to volume.
